# Late recurrence of Cushing disease with *ubiquitin-specific protease 8* mutation 19 years after initial surgery

**DOI:** 10.1210/jcemcr/luag055

**Published:** 2026-04-08

**Authors:** Keisuke Kakizawa, Miho Yamashita, Shu Yanagida, Yuto Kawauchi, Yutaka Oki, Akio Matsushita

**Affiliations:** Second Department of Internal Medicine, Hamamatsu University School of Medicine, Hamamatsu, Shizuoka 431-3192, Japan; International Center, Hamamatsu University School of Medicine, Hamamatsu, Shizuoka 431-3192, Japan; Second Department of Internal Medicine, Hamamatsu University School of Medicine, Hamamatsu, Shizuoka 431-3192, Japan; Second Department of Internal Medicine, Hamamatsu University School of Medicine, Hamamatsu, Shizuoka 431-3192, Japan; Department of Internal Medicine, Hamamatsu Kita Hospital, Hamamatsu, Shizuoka 431-3113, Japan; Second Department of Internal Medicine, Hamamatsu University School of Medicine, Hamamatsu, Shizuoka 431-3192, Japan

**Keywords:** Cushing disease, USP8 gene, recurrence

## Abstract

Recurrence after successful transsphenoidal surgery (TSS) for Cushing disease (CD), caused by adrenocorticotropic hormone (ACTH)-secreting pituitary adenomas, remains a major clinical concern. Somatic mutations in the *ubiquitin-specific protease 8 (USP8)* gene have been implicated in corticotroph tumorigenesis and may contribute to recurrence risk. Here, we report a rare case of delayed CD recurrence 19 years after initial surgical remission. A female aged 36 years underwent TSS for CD with typical clinical and biochemical evidence of hypercortisolism. Magnetic resonance imaging showed a 10 mm pituitary adenoma, and histopathology confirmed an ACTH-producing tumor with a low Ki-67 index (<1%). She achieved long-term postoperative remission. At age 55 years, she developed biochemical and radiological findings consistent with recurrence. Inferior petrosal sinus sampling confirmed localization of the ACTH-secreting lesion to the pituitary gland, and repeat surgery was performed. The recurrent tumor demonstrated a high Ki-67 index. Retrospective Sanger sequencing of the initial tumor identified a *USP8* c.2159C>G p.(Pro720Arg) mutation, a common variant in CD. This case underscores the potential role of *USP8* mutations in long-latency recurrence and highlights the value of genetic profiling. Lifelong endocrine follow-up may be warranted in patients with *USP8*-mutated CD, even after extended postoperative remission.

## Introduction

Cushing syndrome is characterized by chronic glucocorticoid excess (hypercortisolemia) and distinct clinical features, such as moon face. Its etiology is broadly categorized into adrenocorticotropic hormone (ACTH)-dependent and ACTH-independent forms. Among ACTH-dependent causes, Cushing disease (CD), resulting from an ACTH-secreting pituitary adenoma, is the most common. Transsphenoidal surgery (TSS) is the first-line treatment; however, recurrence remains a significant concern, with reported rates ranging from 5% to 35% [1].

Somatic *USP8* mutations occur in up to 30% of corticotroph tumors [2] and may promote tumor growth through epidermal growth factor receptor (EGFR) pathway activation [3]. Recent meta-analyses also indicate that patients with *USP8*-variant corticotroph tumors have a higher recurrence risk [2]. Here, we describe a case of CD with long-term remission followed by recurrence, in which a *USP8* mutation was identified.

## Case presentation

The patient was a female aged 36 years with primary hypothyroidism, hypertension, dyslipidemia, osteoporosis, multiple rib fractures, and postoperative uterine fibroids. She had no history of smoking or alcohol use and was a full-time homemaker. She had previously undergone surgery for uterine fibroids, and although her menstrual cycles were somewhat irregular, she did not experience amenorrhea. She participated in annual health check-ups, and at the age of 36 years, she was diagnosed with hypertension and dyslipidemia. Further evaluation revealed typical signs of Cushing syndrome, including moon face, central obesity, and a buffalo hump, but there were no abdominal violaceous striae. A 75-g oral glucose tolerance test confirmed the absence of diabetes.

## Diagnostic assessment

**Figure 1:**
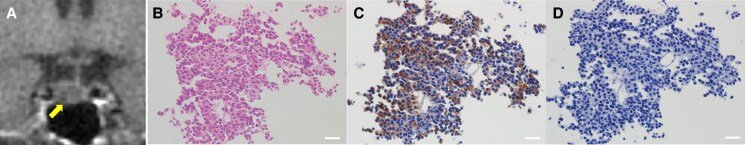
Radiological and pathological findings at the initial surgery. T1-weighted MRI showed a 10 × 5 × 5 mm pituitary adenoma (A, arrow). Hematoxylin and eosin staining (B) and immunohistochemical staining for ACTH (C) and Ki-67 (D) confirmed an ACTH-producing pituitary adenoma with low proliferative activity. Scale bars, 20 μm.

Blood tests confirmed elevated plasma ACTH and serum cortisol (ACTH 75.0 pg/mL [SI: 16.5 pmol/L], ref: 7.2-63.3 pg/mL [SI: 1.6-14.1 pmol/L]; cortisol 21.4 μg/dL [SI: 590.4 nmol/L], ref: 7.1-19.6 μg/dL [SI: 195.9-540.8 nmol/L]). Diurnal variation was lost (16.6 μg/dL [SI: 458.0 nmol/L] at 11:00 Pm). Cortisol was inadequately suppressed on the 1 mg dexamethasone suppression test (DST; 13.9 μg/dL [SI: 383.5 nmol/L]) but showed adequate suppression on the 8 mg DST (2.5 μg/dL [SI: 69.0 nmol/L]). Corticotropin-releasing hormone (CRH) stimulation produced a marked ACTH increase (477%). Pituitary magnetic resonance imaging (MRI) showed a 10 × 5 × 5 mm adenoma (Fig. 1A). These findings supported a diagnosis of CD.

## Treatment

**Figure 2:**
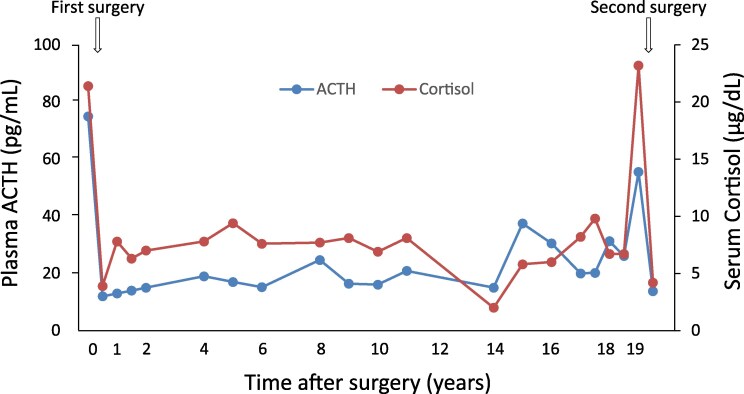
Clinical course of plasma ACTH and serum cortisol levels after surgery demonstrated sustained long-term postoperative remission for 19 years.

**Figure 3:**
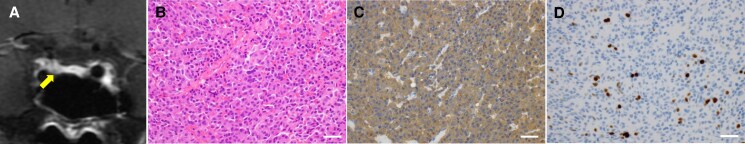
Radiological and pathological findings at recurrence. T1-weighted MRI demonstrated a small nodular lesion on the right side of the pituitary gland (A, arrow). HE staining (B) and immunohistochemical staining for ACTH (C) and Ki-67 (D) identified a densely granulated corticotroph adenoma with high proliferative activity. Scale bars, 50 μm.

The patient underwent TSS. Immunohistochemistry confirmed an ACTH-producing pituitary adenoma with a Ki-67 index of < 1% (Fig. 1B–1D). By postoperative day 7, serum cortisol decreased to 2.8 μg/dL [SI: 77.3 nmol/L]. Over 19 months, postoperative glucocorticoid replacement therapy was gradually tapered and discontinued. Thereafter, the patient experienced long-term remission; however, 19 years later, both plasma ACTH and cortisol levels increased again (ACTH 55.5 pg/mL [SI: 12.2 pmol/L]; cortisol 23.2 μg/dL [SI: 640.0 nmol/L]) (Fig. 2). At the time, moon face was observed; however, no other typical physical signs of Cushing syndrome were evident, nor was there any apparent deterioration of metabolic abnormalities. MRI revealed a small right-sided pituitary lesion (Fig. 3A). ACTH increased following CRH and desmopressin (245% and 417%). Cortisol after the 1 mg DST was 18.6 μg/dL [SI: 513.1 nmol/L], while the 8 mg DST produced adequate suppression (2.4 μg/dL [SI: 66.2 nmol/L]). Although changes in peripituitary blood flow after the initial TSS may compromise the reliability of inferior petrosal sinus (IPS) sampling, previous studies have shown that IPS sampling remains useful for confirming pituitary ACTH hypersecretion in postoperative non-remission cases with non-diagnostic MRI findings [4]. Accordingly, IPS sampling was performed in this case. IPS sampling confirmed pituitary origin (right IPS/peripheral ratio: 17.6) (Table 1). Recurrent CD was diagnosed, and repeat TSS was performed. Histopathology showed a densely granulated corticotroph adenoma with a Ki-67 index of 8.4% (Fig. 3B–3D). Although the early morning serum cortisol level decreased to 4.2 μg/dL [SI: 115.9 nmol/L] after surgery (Fig. 2), this reduction was considered insufficient to ensure long-term remission. Furthermore, the resected tumor showed a high Ki-67 index, suggesting potentially aggressive behavior of the tumor. Consequently, radiosurgery was administered.

**Table 1:** Results of ACTH in inferior petrosal sinus (IPS) sampling before and after intravenous administration of 100 mcg CRH

IPS sampling	Right IPS	Left IPS	Cavernous sinus: right	Cavernous sinus: left	Peripheral vein
Baseline	263 pg/mL (58.4 pmol/L)	44.8 pg/mL (10.0 pmol/L)	1838pg/mL (408.4 pmol/L)	40.8 pg/mL (9.1 pmol/L)	42.7 pg/mL (9.5 pmol/L)
+5 min CRH	3196 pg/mL (710.2 pmol/L)	205 pg/mL (45.6 pmol/L)			182 pg/mL (40.4 pmol/L)
+10 min CRH	1830pg/mL (406.6 pmol/L)	319 pg/mL (70.9 pmol/L)			316 pg/mL (70.2 pmol/L)
+15 min CRH	737 pg/mL (163.8 pmol/L)	391 pg/mL (86.9 pmol/L)			372 pg/mL (82.7 pmol/L)

Abbreviations: ACTH, adrenocorticotropic hormone; CRH, corticotropin-releasing hormone.

## Outcome and follow-up

**Figure 4:**
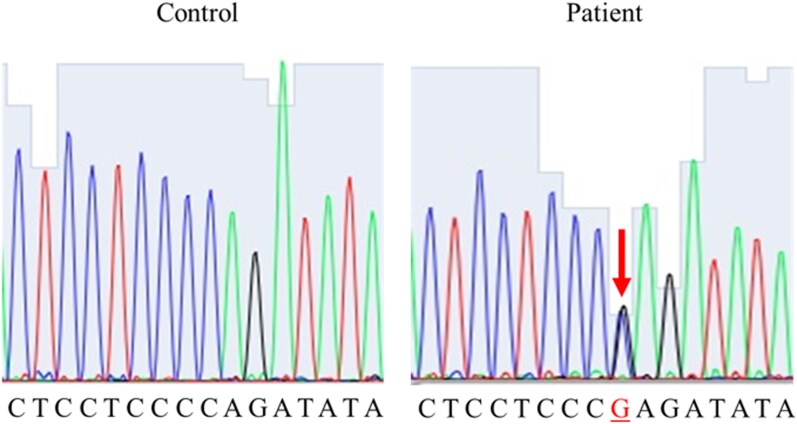
*USP8* Sanger sequencing. The c.2159C>G p.(Pro720Arg) mutation is shown in the initial surgical specimen of the patient.

No further recurrence has been observed during 3.5 years of follow-up. To investigate the cause of recurrence after such prolonged remission, genetic analysis of the original tumor was performed. Because *USP8* exon 14 mutations are common in CD, this region was examined using polymerase chain reaction primers previously described (Forward: 5′-CTTGACCCAATCACTGGAAC-3′; Reverse: 5′-TTACTGTTGGCTTCCTCTTCTC-3′) [5, 6]. Analysis of the initial specimen identified a *USP8* missense mutation, c.2159C>G p.(Pro720Arg) (Fig. 4).

## Discussion

This case illustrates a rare CD recurrence nearly 2 decades after surgery. Reported relapse intervals range from 3 to 158 months (mean 51 months), with about 50% of recurrences occurring within 50 months [7]. A previous report described a patient with cyclic Cushing syndrome who relapsed after 19 years, likely triggered by COVID-19 infection and associated interleukin-6 elevation and high-dose glucocorticoid therapy [8]. In contrast, no infectious or psychosocial stressors were present in our case. Several factors have been associated with remission, including preoperative MRI tumor visualization [9], duration of postoperative glucocorticoid replacement [10], postoperative cortisol levels [11], and *USP8* mutations [12].

A meta-analysis by Pérez-Rivas et al reported *USP8* mutations in approximately 31% of corticotroph tumors, associated with younger onset, female predominance, and higher remission and recurrence rates. The c.2159C>G p.(Pro720Arg) variant is one of the most frequent *USP8* mutations (approximately 35.5%) [2]. Similarly, Miao et al found *USP8* mutations in 50% of corticotroph tumors [13]. Although recurrence-free survival differences did not reach statistical significance (*P* = .068), recurrence-free survival tended to be shorter in the mutated group (median 76.7 vs 109.2 months). That study also associated the p.Pro720Arg variant with younger age at diagnosis and a higher rate of macroadenomas. In this case, retrospective analysis confirmed the same mutation, supporting its potential role in the slow but persistent proliferation of minimal residual tissue, likely mediated by EGFR pathway activation [3]. This is consistent with the marked increase in Ki-67 (MIB-1) index at recurrence (8.4% vs <1%). The limited sample size from the second surgery conducted at an external institution precluded molecular re-evaluation of the *USP8* status at the time of recurrence. However, the clinical progression and histological changes were consistent with the continued influence of the mutation. A previous study revealed that gefitinib, an EGFR inhibitor, significantly attenuated ACTH secretion in surgically resected primary *USP8*-mutated corticotroph adenoma cells [14], indicating that EGFR inhibitors could potentially serve as a future therapeutic option for *USP8*-mutated CD. Recent work also suggests a regulatory relationship between USP8 activity and Wnt signaling [15]. Although pituitary tumors with USP8 mutations are often small, this pathway could still influence recurrence.

In a study by Zhou et al of 107 patients with CD, recurrence occurred more frequently in those with *USP8* mutations than in patients with wild-type tumors (26.5% vs 5.8%, *P* = .009). Multivariate analysis identified *USP8* mutation status, elevated postoperative morning cortisol (>2.5 μg/dL [SI: 69.0 nmol/L]), and incomplete suppression on the 1 mg DST (serum cortisol > 0.78 μg/dL [SI: 21.5 nmol/L]) as independent predictors of 5-year recurrence [16]. The patient discussed here had a *USP8* mutation and a postoperative cortisol level of 2.8 μg/dL [SI: 77.3 nmol/L], both indicating elevated risk. In *USP8* mutation–positive cases, although intensive follow-up may be appropriate when remission criteria are met, additional therapies such as radiotherapy may be considered to reduce the risk of recurrence if there is any suspicion of residual disease, including insufficient postoperative cortisol suppression, as observed in the present case.

Overall, these findings strengthen the role of *USP8* genotyping as a prognostic tool for long-term CD recurrence. Given the unusually long latency in this case (19 years), lifelong follow-up appears warranted in patients with *USP8*-mutated CD, even when remission is initially sustained.

## Learning points

- *USP8* gene mutations, including c.2159C>G p.(Pro720Arg), are associated with an increased risk of CD recurrence, likely through activation of EGFR signaling.
- Lifelong monitoring may be necessary in *USP8*-mutated CD because very late recurrence can occur even after prolonged remission, as demonstrated in the present case.
- Genetic analysis of surgical tumor specimens, including retrospective evaluation, can provide meaningful prognostic information and support long-term management planning in CD.

## Contributors

All authors made individual contributions to authorship. K.K., M.Y., Y.K., and Y.O. contributed to the diagnosis and management of the patient. S.Y. and A.M. contributed to the acquisition and interpretation of the clinical and pathological data. All authors reviewed and approved the final draft.

## Data Availability

Original data generated and analyzed for this case report are included in this published article.
